# Development of a Heat-Killed *fbp1* Mutant Strain as a Therapeutic Agent To Treat Invasive Cryptococcus Infection

**DOI:** 10.1128/spectrum.04955-22

**Published:** 2023-01-31

**Authors:** Yina Wang, Keyi Wang, Amariliz Rivera, Chaoyang Xue

**Affiliations:** a Public Health Research Institute, New Jersey Medical School, Rutgers University, Newark, New Jersey, USA; b Graduate School of Biomedical Sciences, New Jersey Medical School, Rutgers University, Newark, New Jersey, USA; c Department of Pediatrics, New Jersey Medical School, Rutgers University, Newark, New Jersey, USA; d Center for Immunity and Inflammation, New Jersey Medical School, Rutgers University, Newark, New Jersey, USA; e Department of Microbiology, Biochemistry and Molecular Genetics, New Jersey Medical School, Rutgers University, Newark, New Jersey, USA; Stony Brook University

**Keywords:** *Cryptococcus neoformans*, vaccine, protective immunity, F-box protein, Fbp1, therapeutic agent, therapeutic vaccine

## Abstract

In previous studies, we determined that the F-box protein Fbp1, a subunit of the SCF(Fbp1) E3 ligase in Cryptococcus neoformans, is essential for fungal pathogenesis. Heat-killed *fbp1Δ* cells (HK-fbp1) can confer vaccine-induced immunity against lethal challenge with clinically important invasive fungal pathogens, e.g., C. neoformans, C. gattii, and Aspergillus fumigatus. In this study, we found that either CD4^+^ T cells or CD8^+^ T cells were sufficient to confer protection against lethal challenge by C. neoformans in HK-fbp1-induced immunity. Given the potent effect of HK-fbp1 as a preventative vaccine, we further tested the potential efficacy of administering HK-fbp1 cells as a therapeutic agent for treating animals after infection. Remarkably, administration of HK-fbp1 provided robust host protection against preexisting C. neoformans infection. The mice infected with wild-type H99 cells and then treated with HK-fbp1 showed significant reduction of fungal burden in the infected lung and no dissemination of fungal cells to the brain and spleen. We find that early treatment is critical for the effective use of HK-fbp1 as a therapeutic agent. Immune analysis revealed that early treatment with HK-fbp1 cells elicited Th1-biased protective immune responses that help block fungal dissemination and promote better host protection. Our data thus suggest that HK-fbp1 is both an effective prophylactic vaccine candidate against C. neoformans infection in both immunocompetent and immunocompromised populations and a potential novel therapeutic strategy to treat early-stage cryptococcosis.

**IMPORTANCE** Invasive fungal infections, e.g., cryptococcosis, are often life threatening and difficult to treat with very limited therapeutic options. There is no vaccine available in clinical use to prevent or treat fungal infections. Our previous studies demonstrated that heat-killed *fbp1*Δ cells (HK-fbp1) in Cryptococcus neoformans can be harnessed to confer protection against a challenge by the virulent parental strain, even in immunocompromised animals, such as ones lacking CD4^+^ T cells. In this study, we further determined that T cells are required for vaccine-induced protection against homologous challenge and that either CD4^+^ or CD8^+^ cells are sufficient. This finding is particularly important for the potential utility of this vaccine candidate in the context of HIV/AIDS-induced immune deficiency, the main risk factor for cryptococcosis in humans. Furthermore, in addition to the utility of HK-fbp1 as a prophylactic vaccine, we found that HK-fbp1 administration can inhibit disease dissemination when animals are treated at an early stage during Cryptococcus infection. Our findings could significantly expand the utility of HK-fbp1 not only as a prophylactic vaccine but also as a novel therapy against cryptococcosis. In all, our studies showed that the HK-fbp1 strain can be used both preventively and therapeutically to elicit robust host protection against cryptococcosis.

## INTRODUCTION

Invasive fungal infections are emerging diseases that kill over 1.5 million people annually worldwide (1). As the immunocompromised population increases due to HIV infection, aging, and immunosuppressive treatments, including for transplantation and chemotherapy, etc., the incidence of invasive fungal infections is expected to rise further (1). Because fungi are eukaryotes that share much of their cellular machinery with host cells, our armamentarium of antifungal drugs is highly limited, with only three classes of antifungal drugs available (2). Among them, polyenes are toxic, triazoles are fungistatic, and echinocandins have no effect against cryptococcal infections (3). With limited drug options and the emergence of drug resistance, there is an urgent need to develop new strategies to prevent and treat invasive fungal infections to ease the public health burden they cause. For many other infectious diseases caused by viruses and bacteria, vaccines have had a transformative impact on human health and wellbeing worldwide (4). There have been numerous studies focusing on identifying fungal mutants and antigenic factors for potential fungal vaccine development, and a vaccine against candidiasis has completed a phase II clinical trial (4–7). However, despite heroic efforts, there are currently no vaccines in clinical use to combat fungal infections.

Cryptococcus neoformans is a globally distributed pathogen that causes most cases of fungal meningitis in patients with HIV/AIDS, and it is responsible for more than 180,000 deaths annually (8). People with T cell immunodeficiency, such as HIV/AIDS patients, are highly susceptible to Cryptococcus infection, indicating the importance of cell-mediated immunity in host protection (7, 9). A wild-type C. neoformans strain, H99, expressing interferon gamma (IFN-γ) (H99γ), has been shown to induce high Th1 immune responses and to provide full protection against virulent wild-type challenge; these findings also demonstrate the importance of cell-mediated immunity (10). Indeed, C. neoformans mutant strains capable of inducing a highly protective Th1 response have also been reported. Several mutant strains, such as the strain overexpressing the transcription factor Znf2 (ZNF2^OE^) (11), a chitosan-deficient strain (*cda1*Δ*2*Δ*3*Δ) (12), and a mutant lacking sterylglucosidase (*sgl1*Δ) (13), have been identified as having increased immunogenicity in murine models. Their potential in vaccine development has also been proposed, and encouraging data have been reported (4, 7, 12, 14, 15). In addition to whole-cell vaccine strategies, simplified fungal antigenic factors, such as glucan particles, have also been identified for vaccine development (6, 16, 17). All of these exciting developments suggest that a vaccine against Cryptococcus or other invasive fungal infections may be feasible.

In previous studies, we identified an F-box protein, Fbp1, a subunit of the SCF(Fbp1) E3 ligase, and characterized the importance of SCF(Fbp1) E3 ligase-mediated proteolysis in fungal development and virulence (18, 19). We recently showed that an *fbp1*Δ mutant can trigger superior Th1 protective immunity in a CCR2^+^ monocyte-dependent manner and found that both innate and adaptive immunity are involved in the host protection against *fbp1*Δ infection (20). Heat-killed *fbp1*Δ cells (HK-fbp1) also induce strong Th1 responses, and mice vaccinated with HK-fbp1 cells show protection against challenge with the virulent parental strain (20, 21). These data indicate that the heat-killed *fbp1*Δ mutant may have potential for further development as a vaccine. Our studies also demonstrated that HK-fbp1 vaccination can trigger protection not only against its parental strain but also against additional invasive fungal infections, including those caused by C. neoformans, Cryptococcus gattii, and Aspergillus fumigatus. Importantly, HK-fbp1-induced protection against C. neoformans is effective even in immunocompromised hosts, including animals lacking CD4^+^ T cells (21). We found that CD4^+^ T cell-depleted mice had increased CD8^+^ T cell recruitment and increased Th1 cytokine production to compensate for the loss of CD4^+^ T cells (21).

In this study, we further determined that the presence of either CD4^+^ T cells or CD8^+^ T cells is sufficient for complete protection against challenge with wild-type H99 in mice previously vaccinated with HK-fbp1. Moreover, we investigated the potential therapeutic value of HK-fbp1 cells in treating infected mice. Remarkably, administration of HK-fbp1 provided robust host protection against preexisting C. neoformans H99 infection. We found that early administration is critical for the therapeutic efficacy induced by HK-fbp1 cells. We determined that treatment with HK-fbp1 cells during the early stage of Cryptococcus infection promotes enhanced Th1 immune responses and elicits better host protection. In aggregate, our data indicate that the HK-fbp1 strain has the potential to be a suitable prophylactic vaccine candidate against invasive fungal infections and, also, a potential therapeutic agent for early-stage cryptococcosis.

## RESULTS

### CD4^+^ or CD8^+^ T cells are sufficient for full protection against wild-type H99 challenge in HK-fbp1-vaccinated mice.

**FIG 1:**
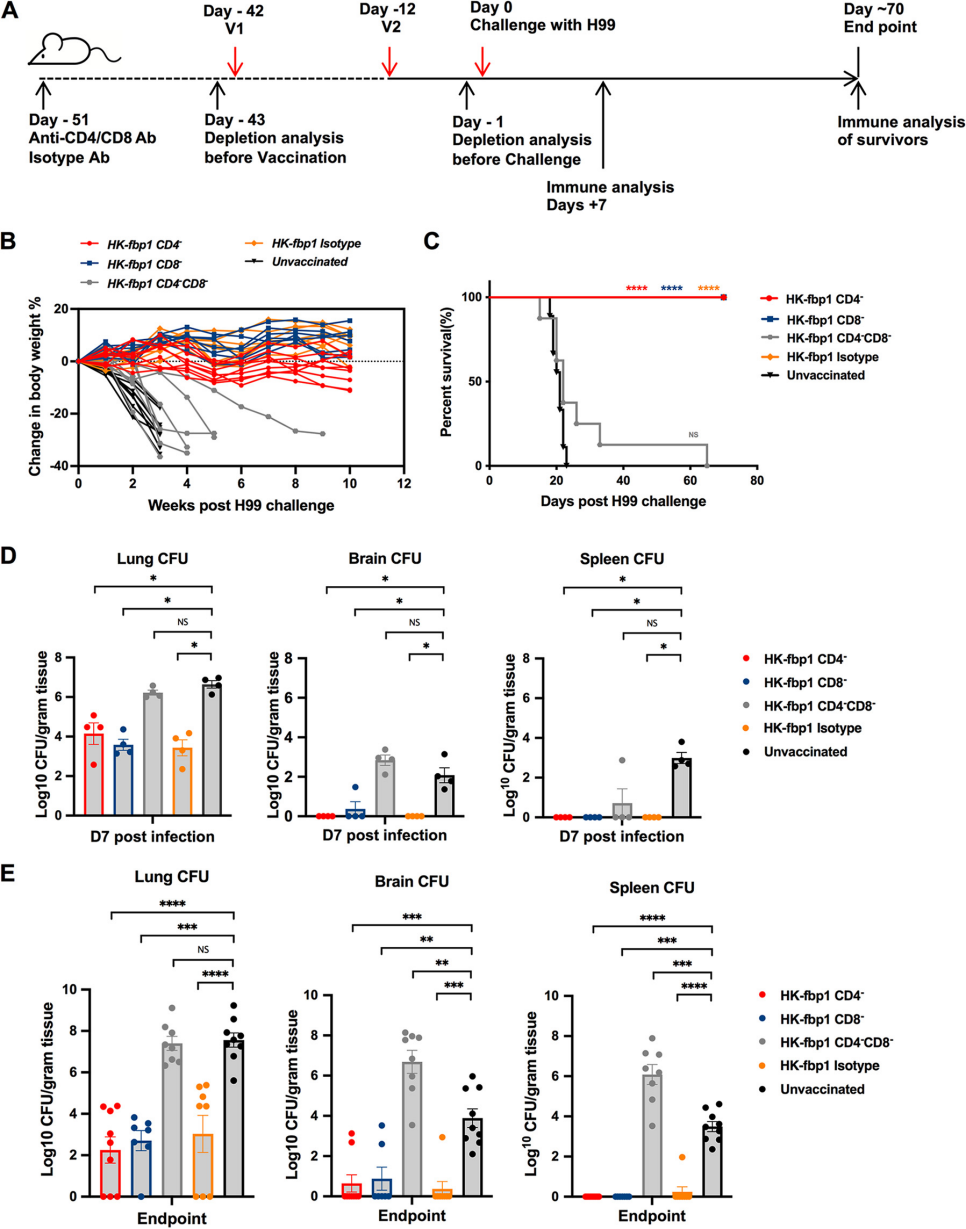
Animals with depletion of either CD4^+^ T cells or CD8^+^ T cells remain fully protected by HK-fbp1 vaccination. (A) Strategy of vaccination. Depletion of CD4^+^ T cells and/or CD8^+^ T cells was accomplished by injecting anti-CD4 antibody (GK1.5, rat IgG2b) or anti-CD8 antibody (116-13.1) starting 9 days prior to the first vaccination and weekly thereafter during the course of the experiment. (B) Dynamic changes of body weight in vaccinated and unvaccinated animals after challenge with 1 × 10^4^ H99 cells. All live mice from each group were weighed, and their average weight changes are presented. (C) Survival curves of CBA/J mice vaccinated with 5 × 10^7^ HK-fbp1 and challenged with 1 × 10^4^ H99 cells. ****, *P* < 0.0001 (determined by log rank [Mantel-Cox] test). (D) Fungal burdens in the lungs, brains, and spleens of vaccinated animals infected with 1 × 10^4^ H99 cells at day 7 postinfection and fungal burdens in unvaccinated control animals. Each symbol represents one mouse. Bars represent the mean values ± standard errors of the means. *, *P* < 0.05; ns, not significant (determined by Mann-Whitney test). (E) Fungal burdens in the lungs, brains, and spleens of vaccinated animals infected with 1 × 10^4^ H99 cells at the endpoint and fungal burdens in unvaccinated control animals. Bars represent the mean values ± standard errors of the means. ****, *P* < 0.0001; ***, *P* < 0.001; **, *P* < 0.01; ns, not significant (determined by Mann-Whitney test).

Individuals with immunodeficiency, such as impaired T cell function in HIV/AIDS patients, are highly susceptible to C. neoformans infection, suggesting the importance of T cells in defense against cryptococcosis (9). Our previous studies demonstrated that the *fbp1*Δ mutant elicited superior protective Th1 host immunity in the lungs and that the enhanced immunogenicity of heat-killed *fbp1*Δ (HK-fbp1) yeast cells can be harnessed to confer protection against a subsequent infection with the virulent parental strain in immunocompetent or CD4^+^ T cell-deficient hosts (20, 21). Given the clinical significance of T cell deficiency to the susceptibility to cryptococcosis in patients, it is also critical to know whether host protection can be established following HK-fbp1 vaccination in both CD4^+^ and CD8^+^ T cell-deficient hosts. Therefore, we further examined the potential vaccine protection in mice depleted of CD4^+^ and/or CD8^+^ T cells. An animal model of T cell deficiency was achieved by the administration of 200 μg/mouse anti-CD4 antibody and/or 100 μg/mouse anti-CD8 antibody 9 days prior to the first vaccination and weekly thereafter during the course of the experiment (Fig. 1A). Efficient depletion was confirmed by measuring the prevalence of CD4^+^ T cells and CD8^+^ T cells in blood samples by flow cytometry on the day before the first vaccination (day −43) and the day before challenge (day −1) (Fig. S1 in the supplemental material). We tracked the changes in animal body weight weekly throughout the experiment. We noticed that CD4^+^ T cell-depleted, CD8^+^ T cell-depleted, and isotype control animals challenged with H99 cells maintained or increased body weight over time, while the CD4^+^ and CD8^+^ T cell double-depleted mice and unvaccinated ones lost weight rapidly following infection (Fig. 1B). Consistent with animal body weight changes, mice administered anti-CD4 antibody, anti-CD8 antibody, or isotype control antibody survived for over 2 months after challenge with H99. Thus, HK-fbp1-vaccinated animals depleted of CD4^+^ or CD8^+^ T cells were fully protected against H99 challenge (Fig. 1C and Fig. S1). The difference between the median survival times for vaccinated mice with CD4^+^ and CD8^+^ double T cell depletion and unvaccinated mice was not statistically different, suggesting that vaccination does not work in mice that lack both T cell subsets (Fig. 1C).

At 7 days postchallenge, five mice from each group were sacrificed and examined for fungal burdens in lungs, brains, and spleens. For CD4^+^ T cell-depleted or CD8^+^ T cell-depleted animal groups and the isotype control group, no CFU were detected in brains and spleens of all but one infected mouse, and significantly lower CFU counts were detected in the lungs of infected animals than in the lungs of nonvaccinated animals. The fungal burdens in the CD4^+^ and CD8^+^ T cells double-depleted mice were similar to those in the unvaccinated control group, indicating that the presence of either CD4^+^ or CD8^+^ T cells is necessary and also sufficient to induce protective immunity against C. neoformans challenge (Fig. 1D). We also analyzed the endpoint (day 70) fungal burdens in these animal groups and observed significantly reduced fungal burdens in the lungs of vaccinated animals, except for the CD4^+^ and CD8^+^ T cell double-depleted mice. Most of the brains and spleens of immunized animals were cleared of H99 cells during the period of this experiment, suggesting that immunization helped CD4^+^ or CD8^+^ T cell-deficient animals clear or restrict Cryptococcus cell proliferation (Fig. 1E). Altogether, our studies showed that the HK-fbp1 vaccination-induced protection is dependent on T cells and that the vaccine remains effective in immunocompromised hosts that lack either CD4^+^ or CD8^+^ T cells, but not both.

### Robust cytokine production by either CD4 or CD8 T cells underlies HK-fbp1-induced vaccine protection.

**FIG 2:**
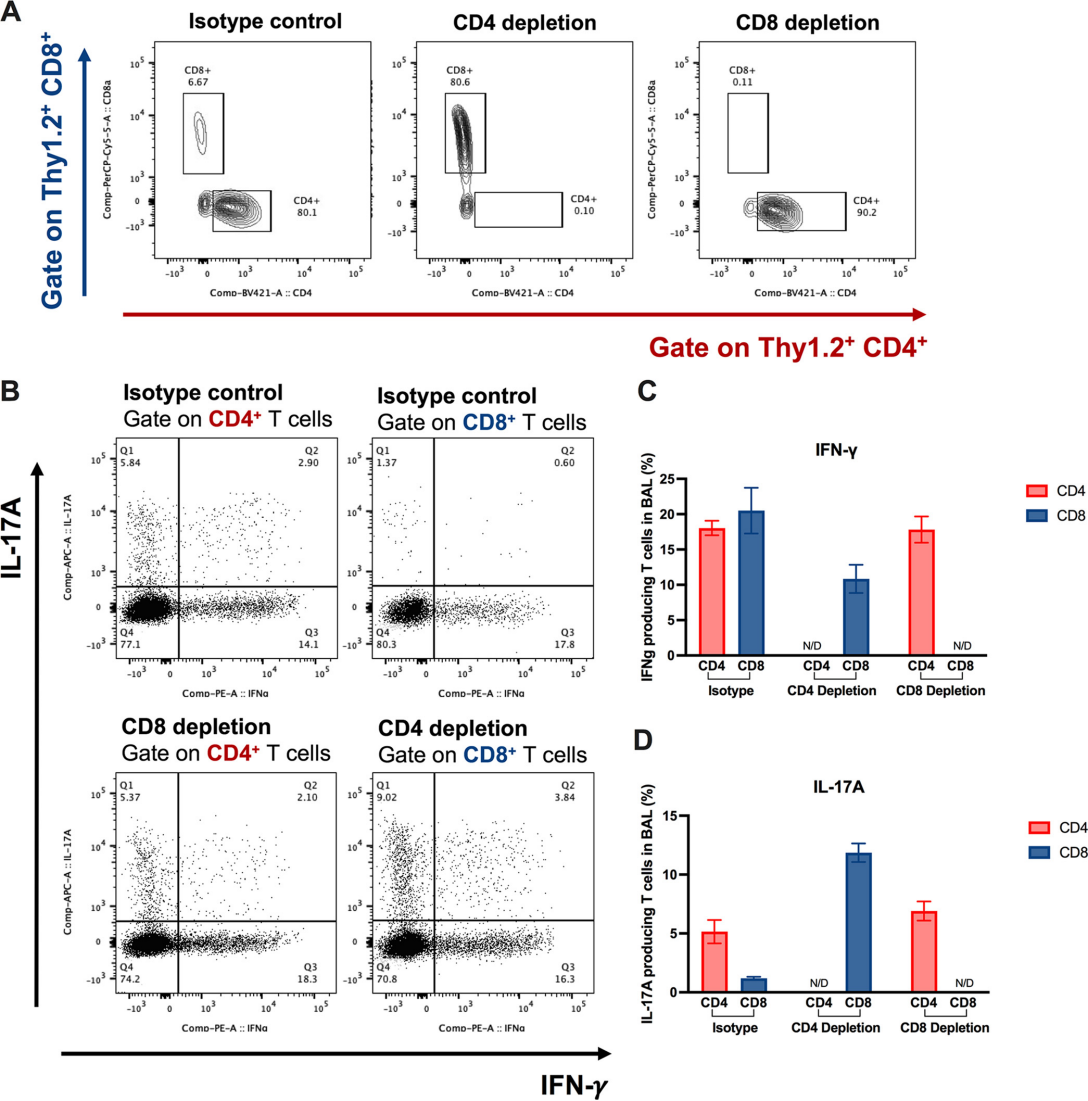
Role of CD4^+^ and CD8^+^ T cells and enhanced Th1 and Th17 T cell responses during the induction of protective immunity by HK-fbp1 in immunodeficient animal model. (A) Representative FACS plots of CD4^+^ T cells and CD8^+^ T cells in the BALF samples of isotype control mice (left), CD4^+^ T cell-depleted mice (middle), and CD8^+^ T cell-depleted mice (right). (B) Representative FACS plots of cytokine production gate on CD4^+^ T cells or CD8^+^ T cells in isotype control mice (top), CD4-depleted mice (bottom), and CD8-depleted mice (bottom) at day 7 postinfection. (C and D) Cytokine expression analyzed by ICCS. Plots of cytokine production in CD4^+^ T cells gated as Thy1.2^+^ CD4^+^ CD8^−^ T cells. Plots of cytokine production in CD8^+^ T cells gated as Thy1.2^+^ CD4^−^ CD8^+^ T cells. The frequencies of IFN-γ-producing (C) and IL-17A-producing (D) CD4^+^ or CD8^+^ T cells in BALF samples were analyzed as shown. comp, compensation.

To understand how protection is established in either CD4^+^ or CD8^+^ T cell-depleted mice, we examined the immune responses of the remaining CD4^+^ or CD8^+^ T cell populations in vaccinated and unvaccinated mice at day 7 postchallenge and also at the endpoint of the experiment (day 70). At day 7 postinfection, we found that while the isotype control-treated mice had a significant increase in the number of CD4^+^ T cells in the infected lungs, CD4^+^ T cells were not detectable in the bronchoalveolar lavage fluid (BALF) samples of CD4^+^ T cell-depleted mice, and CD8^+^ T cells were not detectable in the lungs of CD8^+^ T cell-depleted mice, as expected (Fig. 2A). CD4^+^ or CD8^+^ T cell depletion was maintained weekly until the endpoint of the experiment (day 70). We examined T cells in lungs, BALF samples, and lung-draining (mediastinal) lymph nodes (mLN) at the endpoint and confirmed that CD4^+^ or CD8^+^ T cells were completely depleted throughout the whole experiment (Fig. S2).

Previously, we determined that IFN-γ is a critical mediator of vaccine-induced protection in this model (20, 21). Mice defective in IFN-γ responsiveness were not protected after HK-fbp1 vaccination and were as susceptible to infection as unvaccinated mice (21). Therefore, to understand how remaining T cell populations protected vaccinated mice against challenge with H99, we examined the cytokine responses of the remaining CD4^+^ T cells or CD8^+^ T cells in the airway at day 7 postinfection by intracellular cytokine staining. Our data show that CD8^+^ T cells produced IFN-γ and interleukin-17A (IL-17A) in CD4^+^ T cell-depleted mice (Fig. 2). In the isotype control-treated mice, these protective cytokines were mainly produced by CD4^+^ T cells (Fig. 2). Meanwhile, in the CD8^+^ T cell-depleted mice, CD4^+^ T cells were robust producers of protective cytokines (Fig. 2B to D). These results suggest that CD8^+^ T cells are able to produce protective cytokines, which can compensate for the lack of CD4^+^ T cells. Our findings thus indicate that CD4^+^ T cells or CD8^+^ T cells are sufficient to confer protection against challenge with H99 in HK-fbp1-vaccinated mice due to their ability to produce protective IFN-γ and IL-17.

### Treatment of C. neoformans H99-infected mice with HK-fbp1 vaccine inhibits fungal dissemination.

**FIG 3:**
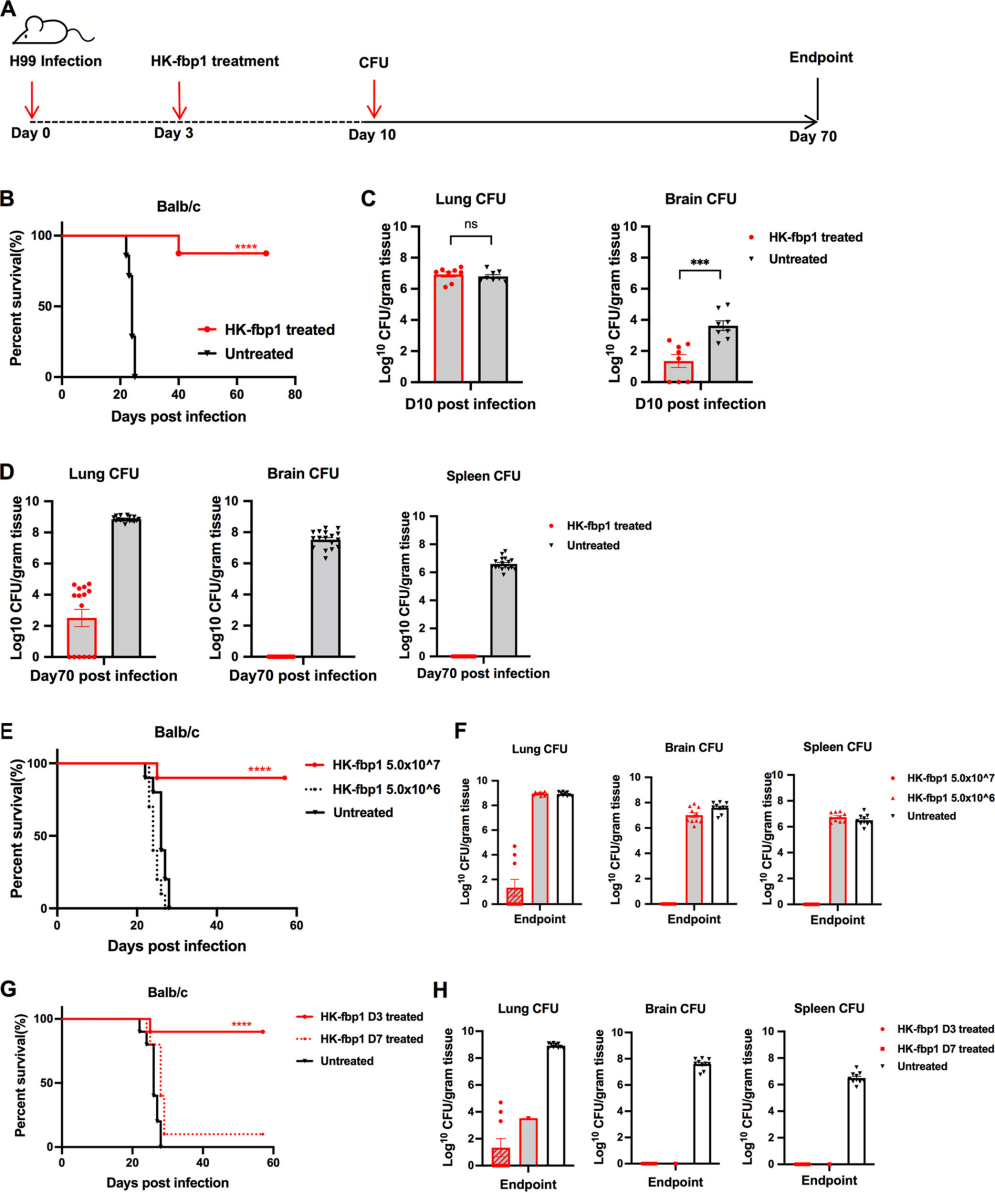
HK-fbp1 treatment of mice infected by C. neoformans H99 blocked fungal dissemination to prevent lethal infection. (A) Scheme of HK-fbp1 treatment strategy. (B) Survival curves of HK-fbp1-treated BALB/c mice challenged with C. neoformans H99. ****, *P* < 0.0001 (determined by log rank [Mantel-Cox] test). (C) Fungal burdens in the infected lungs, brains, and spleens at day 7 posttreatment. Each symbol represents one mouse. Bars represent the mean values ± standard errors of the means. ***, *P* < 0.001; ns, not significant (determined by Mann-Whitney test). (D) Fungal burdens in the infected lungs, brains, and spleens at the end of the experiment (~70 days postchallenge). Each symbol represents one mouse. (E) Survival curves of mice prechallenged by infection with 10^4^ wild-type H99 cells and treated with different doses of HK-fbp1 at day 3 postinfection. Eight to 10 female BALB/c mice were used for each group. ****, *P* < 0.0001 (determined by log rank [Mantel-Cox] test). (F) Fungal burdens in the lungs, brains, and spleens of HK-fbp1-treated survivors at the end of the experiment and fungal burdens of untreated control mice. (G) Survival curves of mice prechallenged with 10^4^ wild-type H99 cells at 3 days and 7 days before treatment with the high dose of HK-fbp1. ****, *P* < 0.0001 (determined by log rank [Mantel-Cox] test). (H) Fungal burdens in the lungs, brains, and spleens of the surviving mice at the end of the experiment and fungal burdens in untreated mice.

A therapeutic vaccine is one in which the vaccine is used after infection occurs, aiming to induce anti-infective immunity to alter the course of disease (22). It works by activating the host immune system to fight an infection. Since the HK-fbp1 vaccine candidate confers high protection against Cryptococcus neoformans infection prophylactically by inducing T cell-dependent protective immunity, we asked whether HK-fbp1 could be applied as a therapeutic agent to treat Cryptococcus-infected hosts by boosting their immunity. To test this idea, previously naive mice were infected with 1 × 10^4^ live H99 cells via intranasal inoculation. At day 3 postinfection, the infected mice were treated intranasally with 5 × 10^7^ HK-fbp1 cells. Treated and untreated animals were monitored for survival for up to 70 days (Fig. 3A).

Remarkably, HK-fbp1 treatment induced significant protection against preexisting H99 infection. All untreated mice succumbed to fatal infection at ~20 days, while most treated animals survived for over 70 days following HK-fbp1 treatment at day 3 postinfection (Fig. 3B). We examined the fungal burdens in the lungs, brains, and spleens of the treated and untreated mice at set time points of the experiment. Although no significant difference in fungal CFU was detected between HK-fbp1-treated lungs and the untreated controls at 7 days posttreatment, significantly reduced CFU were detected in most of the brains of treated animals (Fig. 3C). Endpoint fungal burden analysis revealed significantly reduced fungal burdens in the lungs of HK-fbp1-treated animals. Importantly, none of the surviving mice of the treated group displayed extrapulmonary dissemination of H99 cells to the brain and spleen (Fig. 3D). H99 cells were totally cleared from all brains and spleens of treated animals during the period of the experiment, indicating that HK-fbp1 treatment is sufficient to clear H99 cells or restrict them from dissemination, likely by inducing protective immunity. Taken together, our results demonstrate that treatment with the HK-fbp1 vaccine protects animals from preexisting lethal infection and restrains proliferation of wild-type H99 cells in the lung.

### High dose and early treatment are required for the therapeutic efficacy induced by HK-fbp1 against Cryptococcus infection.

Our previous studies revealed that as a prophylactic vaccine, the efficacy of HK-fbp1 vaccine protection in mice was dose dependent (21). Mice immunized with higher doses of vaccine exhibited better protection than those vaccinated with low doses (21). Mice vaccinated with 5 × 10^7^ HK-fbp1 received 100% protection, while no clear protection was observed for mice vaccinated with 5 × 10^6^ HK-fbp1 cells (21). Since our data showed a potential therapeutic value for our vaccine as a treatment for animals with preexisting infection, we examined the potential dose effect of the HK-fbp1 treatment. Mice were intranasally administered different HK-fbp1 inocula (5 × 10^7^ or 5 × 10^6^ cells/mouse) at day 3 postinfection. We did find a dose-dependent efficacy (Fig. 3E). Mice treated with the higher dose of HK-fbp1 exhibited better protection than those treated with the low dose. Mice treated with 5 × 10^7^ HK-fbp1 received ~90% protection, while no clear protection was observed for mice treated with 5 × 10^6^ HK-fbp1 cells (Fig. 3E). Examination of the fungal burden in high-dose-treated mice that survived H99 infection showed that fungal cells were cleared from the brains and spleens, while the lungs had significantly lower fungal loads (Fig. 3F). Overall, our data suggest that a fungal antigenic threshold has to be reached in order to induce efficient protection with HK-fbp1 treatment.

We then tested whether HK-fbp1 treatment remains effective in infected animals with disseminated disease. Since C. neoformans infection typically disseminates at day 7 postinfection intranasally, while it remains as a local lung infection at day 3, we set two treatment time points: 7 days postinfection as long-term infection and 3 days postinfection as short-term infection. We challenged mice at −7 days or −3 days before HK-fbp1 treatment (Fig. 3G). HK-fbp1 treatment at day 3 postinfection conferred ~90% protection, while no clear protection was observed for mice treated at day 7 postinfection (only 10% of mice survived for 70 days). Examination of the fungal burden in mice treated early showed clearance of fungal cells in the brains and spleens, and infected lungs had significantly lower fungal loads (Fig. 3H). Similar results were observed for the one surviving mouse treated at day 7 postinfection (Fig. 3H). Overall, our data indicate that high dose and early treatment are required for efficacy of the HK-fbp1 vaccine in treating Cryptococcus infection.

### Early treatment with HK-fbp1 induces a protective host response and prevents fungal dissemination.

**FIG 4:**
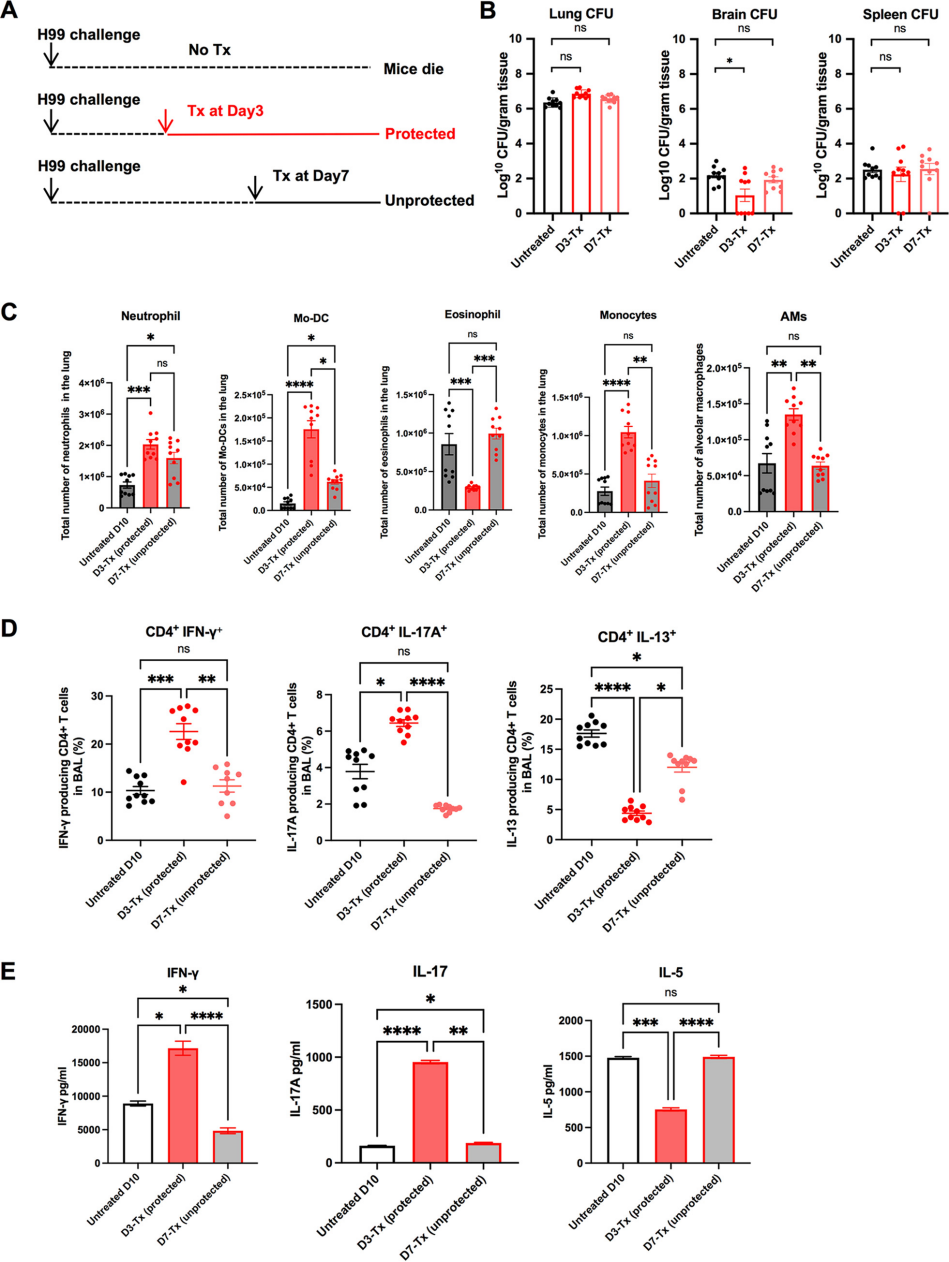
High dose and early intervention are required for the effectiveness of HK-fbp1 treatment against Cryptococcus infection. (A) Scheme of HK-fbp1 treatment strategy. Wild-type BALB/c mice were infected with 1 × 10^4^ H99 at day zero. Mice were treated with 5 × 10^7^ HK-fbp1 on day 3 (early treatment)or day 7 (late treatment). (B) Fungal burdens at day 10 post-H99 infection in the infected lungs, brains, and spleens from untreated mice, day 3 early treatment mice, and day 7 late treatment mice. Each symbol represents one mouse. Bars represent the mean values ± standard errors of the means. *, *P* < 0.05; ns, not significant (determined by Mann-Whitney test). (C) Cellular infiltration of the lungs was analyzed by flow cytometry. Each symbol represents one mouse, and the data are cumulative from two independent experiments. Bars represent the mean values ± standard errors of the means. Each cell population was identified as CD45^+^ DAPI-negative live leukocytes. Mo-DCs were gated as CD11b^+^ Ly6G^−^ Ly6C^hi^ CD11c^+^ MHCII^+^, eosinophils as CD11c^low^ SiglecF^+^, neutrophils as CD11b^+^ Ly6C^int^ Ly6G^hi^, CCR2^+^ monocytes as DAPI^−^ CD45^+^ CD11b^+^ Ly6C^hi^ Ly6G^−^, and alveolar macrophages (AMs) as DAPI^−^ CD45^+^ CD11c^+^ SiglecF^+^. (D) Plots of cytokine production in CD4^+^ T cells gated as Thy1.2^+^ CD4^+^ CD8^−^ T cells in BALF. The frequencies of IFN-γ-, IL-17A-, and IL-13-producing CD4^+^ T cells in BALF were analyzed as shown. (E) CD4^+^ T cells were isolated from the lung-draining lymph nodes of untreated, day 3-treated, and day 7-treated mice. Cytokine secretion in the presence of Cryptococcus antigen was examined by ELISA as described in Materials and Methods. The data shown are cumulative from two independent experiments with five mice per group and are depicted as the mean values ± standard errors of the means. ****, *P* < 0.0001; ***, *P* < 0.001; **, *P* < 0.01; *, *P* < 0.05; ns, not significant (determined by one-way analysis of variance [ANOVA] nonparametric test for multiple comparisons).

Our previous study demonstrated that a strong Th1 immune response developed in mice immunized with HK-fbp1 cells (20, 21). Because mice treated with HK-fbp1 at an early time postinfection showed strong protection and blocked fungal dissemination, we set out to examine the immune response of Cryptococcus-infected mice that were treated with HK-fbp1. Mice were infected with H99 for 3 days or 7 days and then treated with HK-fbp1 (Fig. 4A). Our data showed that at day 10 postinfection, fungal CFU were detected in both day 3-treated mice (early treatment), and day 7-treated (late treatment) mice. Although there was no difference in CFU in the lungs of day 3-treated mice compared to those of day 7-treated mice, the day 3-treated mice showed significantly reduced fungal CFU in infected brains, suggesting an inhibition of fungal dissemination, while the day 7-treated mice showed a brain fungal burden similar to that in untreated mice (Fig. 4B).

We hypothesized that early treatment with HK-fbp1 likely shapes H99 immunogenicity and affects the development of the protective immune response in the host, therefore preventing fungal dissemination in an effective manner. To test this hypothesis, we analyzed the recruitment of immune cells to the lung at day 10 post-H99 infection. Our results showed that early treatment with HK-fbp1 induced an enhanced activation of the innate immune response compared to its activation by the late treatment (Fig. 4C). We observed increased numbers of neutrophils and enhanced monocyte differentiation into monocyte-derived dendritic cells (Mo-DCs) in day 3-treated mice compared to the results for the day 7-treated mice (Fig. 4C). We also detected increased CCR2^+^ monocytes and alveolar macrophages in day 3-treated mice (Fig. 4C). Moreover, we observed a decreased number of eosinophils in day 3-treated mice (Fig. 4C). Eosinophilia is a hallmark of Th2-dominated responses (23, 24), and thus, a reduction in eosinophil numbers is indicative of reduced Th2 responses in HK-fbp1-treated mice.

Previous studies have shown that Th1 (characterized by the production of IFN-γ) and Th17 (characterized by the production of IL-17) CD4^+^ T cells are important in defense against Cryptococcus infection in mouse models (9, 25). In contrast, Th2 responses (characterized by the production of IL-13) are harmful to the host during cryptococcosis (26–28). In our previous study, we determined that monocytes and Mo-DCs are required for protection against *fbp1*Δ infection at least in part via activation of Th1 cells (20). Thus, we hypothesize that the enhanced Mo-DC maturation seen in mice given HK-fbp1 at day 3 after H99 infection (Fig. 4C) helps to promote a protective T cell response. Therefore, we examined the cytokine profiles of CD4^+^ T cells in the airway at day 10 postinfection. We observed that early treatment with HK-fbp1 induced enhanced differentiation of IFN-γ-producing Th1 cells and IL-17A-producing Th17 cells in the airway (Fig. 4D). This increased protective response was accompanied by reduced differentiation of IL-13-producing Th2 cells (Fig. 4D). Reduced Th2 responses in HK-fbp1-treated mice were also indicated by reduced eosinophilia (Fig. 4C). Our aggregate observations suggest that early treatment with HK-fbp1 was able to shape CD4^+^ T cell polarization toward Th1 and Th17 responses and diminished detrimental Th2 differentiation. Similarly, Cryptococcus-specific CD4^+^ T cell responses measured in mLN of mice treated early with HK-fbp1 also produced higher amounts of IFN-γ and IL-17A and lower amounts of IL-5 after *ex vivo* restimulation than were seen in untreated controls or mice treated at day 7 (Fig. 4E). Collectively, these findings indicate that early treatment with HK-fbp1 induced enhanced recruitment of innate immune cells, as well as increased induction of Th1 and Th17 responses and a lower Th2 response. These protective host immune responses helped to promote the containment of fungal cells in the lung and inhibition of fungal dissemination.

## DISCUSSION

In this study, we set out to examine the potential utility of HK-fbp1 as a therapeutic agent to treat cryptococcosis. The rationale for the studies presented stems from our earlier work, where we demonstrated that vaccination with HK-fbp1 can provide potent protection against Cryptococcus infection by inducing a protective T cell response (20, 21). Our results showed that, indeed, the HK-fbp1 vaccine is effective in treating early-stage Cryptococcus infection. However, the therapeutic value is limited when used to treat animals with disseminated cryptococcosis. Together, the results indicate that the T cell-mediated immune response can contain fungal infection from dissemination before it happens.

To understand the role of T cells in HK-fbp1-mediated host protection, we performed a CD4^+^ T cell depletion experiment in a previous vaccination study and found that the lack of CD4^+^ T cells triggered increased CD8^+^ T cell expansion to provide protection (21). In this study, we depleted CD8^+^ T cells and both CD4^+^ and CD8^+^ T cells. While depletion of CD8^+^ T cells did not compromise the protection, we found that the animals depleted of both CD4^+^ and CD8^+^ T cells were no longer protected by the HK-fbp1 vaccine, suggesting that at least one T cell subset is needed to mount effective host protection. This result is consistent with the compensatory role of CD4^+^ and CD8^+^ T cells seen in other models of vaccine protection (29, 30) and is also consistent with a recent report from the *sgl1*Δ vaccination study (14). In that report, Del Poeta and colleagues also found that the presence of either CD4^+^ T cells or CD8^+^ T cells is sufficient to confer protection from the live *sgl1*Δ cell-based vaccine against C. neoformans challenge but that protection is not conferred in the host lacking both CD4^+^ and CD8^+^ T cells. A similar conclusion was also reported in the model of H99γ-based vaccine-induced protection (31). All these studies demonstrated the critical role of T cell-mediated immunity in protection against Cryptococcus infection. Thus, our observations are consistent with findings in other models of vaccination.

Given the importance of CD4^+^ and CD8^+^ T cells in host defense against C. neoformans infection, we investigated the potential of utilizing HK-fbp1 vaccine as a therapeutic agent. We found that, indeed, therapeutic administration of HK-fbp1 can confer potent protection in mice treated early after fungal infection. The observation that the effective therapeutic potential is restricted to early administration is consistent with the interpretation that the mechanism of protection depends on the effective skewing of T cell differentiation toward protective Th1 and Th17 responses. In turn, protective immunity helps by blocking fungal dissemination. While we were testing the therapeutic value of our HK-fbp1 vaccine candidate, an independent study from Del Poeta and colleagues was reported, using the *sgl1*Δ mutant-based vaccine as a therapeutic agent, in which a similar treatment plan was used to treat mice infected by the parental C. neoformans strain (15). In that report, treatment of wild-type infected mice with both heat-killed *sgl1*Δ cells and live *sgl1*Δ cells significantly inhibited the Cryptococcus dissemination, with reduced fungal burdens in the brains of infected mice. Interesting, they also found that early treatment (day 3 postinfection) exhibited a better outcome than later treatment (day 7 postinfection). The similar therapeutic outcomes of these vaccine candidates in treating early stage cryptococcosis suggests that they may share a very similar disease inhibition mechanism that is likely related to their ability to induce strong Th1 protective immunity, although host immune responses were not examined in that study (15).

Previous studies suggest that infection with virulent strains of C. neoformans will induce a high Th2 response, which is detrimental to the host and does not prevent disseminated infection (28, 32, 33). In this study, we observed that mice treated with HK-fbp1 at day 3 after infection exhibited high Th1 and Th17 protective responses, evident by increased production of IFN-γ and IL-17A cytokines and reduced Th2 cytokines (IL-13). In contrast, untreated mice and those treated at day 7 after infection showed lower Th1 responses and robust Th2 responses (IL-5- and IL-13-producing T cells and increased eosinophilia). We conclude that administration of HK-fbp1 at day 7 after infection is unable to significantly alter the fate of T cells that have already committed to a Th2 differentiation program.

Altogether, our study demonstrates that the HK-fbp1 vaccine candidate can not only be utilized as a prophylactic vaccine candidate to prevent multiple important invasive fungal infections but is also effective to treat early-stage Cryptococcus infection as a therapeutic agent. Many people carry Cryptococcus cells in a form of local granuloma without disease symptom (34, 35). A vaccine that can both prevent and treat a preexisting infection may be effective not only prophylactically in the healthy population but also in patients preexposed to fungal cells with only local deposition, by helping prevent these patients from reactivation and the development of disseminated cryptococcosis in the event of immunosuppression or comorbidity. Although the utility of HK-fbp1 as a therapeutic agent may be restricted by the time of treatment and host conditions, further study and product improvement may lead to a more effective agent. In addition, it might be possible to develop a combinational therapy where sequential coadministration of the HK-fbp1 vaccine and antifungal drugs may lead to improved control of fungal burdens and better treatment outcomes even for patients with disseminated diseases.

## MATERIALS AND METHODS

### Animal use and ethics statement.

Female mice with an average weight of 20 to 25 g were used throughout these studies. BALB/c mice were purchased from the Jackson Laboratories, while mice of the CBA/J genetic background were purchased from Envigo. Animal studies were performed at Rutgers University Newark campus animal facility. All studies were conducted following biosafety level 2 (BSL-2) protocols and procedures approved by the Institutional Animal Care and Use Committee (IACUC) and Institutional Biosafety Committee of Rutgers University under protocol 999901066. Animal studies were compliant with all applicable provisions established by the Animal Welfare Act and the Public Health Services (PHS) Policy on the Humane Care and Use of Laboratory Animals.

### Infection with cryptococci.

To prepare fungal cells for infection, overnight cultures of C. neoformans strain H99 were washed three times with 1× phosphate-buffered saline (PBS) buffer and the concentrations of yeast cells were determined by hemocytometer counting. The final fungal concentration was adjusted with 1× PBS to 2 × 10^5^ cells/mL. Each mouse was infected intranasally with 1 × 10^4^ H99 cells in a 50-μL volume after being anesthetized with a mixture of ketamine (12.5 mg/mL) and xylazine (1 mg/mL). After infection, animals were weighed daily and monitored twice daily for progression of disease, including weight loss, gait changes, labored breathing, and fur ruffling. Over the course of the experiments, animals that appeared moribund or in pain were euthanized by CO_2_ inhalation. Survival data from the murine experiments were statistically analyzed between paired groups using the log rank (Mantel-Cox) test with PRISM version 8.0 (GraphPad Software) (*P* values of <0.05 were considered statistically significant). The change in body weight of each animal was calculated as follows: [(weight on day *x* − weight on day 0)/weight on day 0] × 100%. The resulting data were plotted against time. To compare the fungal burdens, infected lungs, brains, and spleens were isolated and homogenized (Ultra-Turrax T8; IKA) in 3 mL cold 1× PBS buffer for 1 min for each type of organ. The tissue suspensions were serially diluted and plated onto yeast extract-peptone-dextrose (YPD) agar medium with ampicillin and chloramphenicol, and colonies were counted after 3 days of incubation at 30°C.

### Vaccination strategy.

The C. neoformans *fbp1*Δ mutant strain was heat killed following a previously described procedure (21). Briefly, fungal cells from YPD overnight cultures were precipitated and washed twice with sterile PBS. The cell suspension with the correct concentration was then aliquoted into Eppendorf tubes and heated on a hot plate at 75°C for 90 min. The viability of the cells following heat treatment was examined by plating the processed cell suspension on YPD agar plates; no colonies were recovered after incubation at 30°C for 3 days. Mice were vaccinated intranasally with 5 × 10^7^ heat-killed fungal cells (HK-fbp1) at day −42 unless otherwise specified. Each group of 8 to 10 mice were vaccinated again with the same dose of heat-killed fungal strains at day −12. A group of unvaccinated mice served as a control. The vaccinated groups and unvaccinated control group were challenged with 1 × 10^4^ live H99 cells via intranasal inoculation. Infected animals were weighed and monitored daily for disease progression, and moribund mice were euthanized. All survivors were euthanized on day 65 after challenge with live H99 cells unless otherwise specified.

### Therapeutic administration strategy.

To evaluate the HK-fbp1 vaccine as a potential therapeutic agent, a heat-killed *fbp1*Δ strain (HK-fbp1) was used to treat mice post-wild-type-H99 challenge. The mice were infected with 1 × 10^4^ live H99 cells via intranasal inoculation at day −7 or day −3 before treatment. One group of mice were then treated intranasally with 5 × 10^7^ HK-fbp1 fungal cells at day zero. Groups of animals treated with HK-fbp1 were sacrificed at day 3 and day 7 posttreatment according to the Rutgers IACUC-approved animal protocol. One group of untreated animals was sacrificed at the same time as a control. For analyzing host immune responses, bronchoalveolar lavage fluid (BALF) samples and lungs were harvested at the designated time points after treatment. Single-cell suspensions of pulmonary cells were prepared for flow cytometric analysis. Infected lungs, brains, and spleens were isolated for fungal burden analysis.

### CD4^+^/CD8^+^ T cell depletion.

Mice were depleted of CD4^+^ T cell subsets or CD8^+^ T cell subsets via intraperitoneal administration of anti-CD4 (GK1.5, rat IgG2b) antibody (catalog number BE0003-1; BioxCell) or anti-CD8 (116-13.1, Lyt2.1) antibody (catalog number BE0118; BioxCell). Each mouse received 200 μg of GK1.5, 100 μg of 116-13.1, or 200 μg of isotype (LTF-2, rat IgG2b) control antibody (catalog number BE0090; BioxCell) in a volume of 200 μL PBS 9 days prior to the first vaccination and weekly thereafter during the observation period. Efficient depletion was confirmed by measuring the prevalence of CD4^+^ or CD8^+^ T cells in blood samples by flow cytometry on the day before the first vaccination (day −43) and the day before challenge (day −1). The depletion was also confirmed by measuring the prevalence of CD4^+^ or CD8^+^ T cells in BALF and lung tissue samples by flow cytometry at the endpoint of the experiment. The anti-CD4 antibody used for flow cytometric analysis binds to an epitope of the CD4 protein at locations distinct from GK1.5 binding. The RM4-4 fluorescein isothiocyanate (FITC) rat anti-mouse CD4 antibody (catalog number 553055; BD Biosciences) and YTS169.4 phycoerythrin (PE) rat anti-mouse CD8 antibody (catalog number MA5-17606;Invitrogen) were used for flow cytometric analysis of blood samples, while the CD4 RM4-5 BV421 rat anti-mouse CD4 antibody (catalog number100543; BD Biosciences) and rat anti-mouse CD8 antibody (catalog number MA5-17606; Invitrogen) were used for flow cytometric analysis of BALF and lung tissue samples.

### Depletion confirmation.

To confirm the efficiency of CD4^+^ or CD8^+^ T cell depletion, animal blood samples were processed for flow cytometry as described previously (21). Blood samples (2 or 3 drops/tail) were collected and placed into 50 μL of heparin (100 USP heparin units/mL) in a 96-well plate. After collection, bleeding was stopped by applying Kwik Stop styptic powder to the end of the tail using a moistened cotton applicator. Blood cells were washed with 150 μL of 1× PBS and resuspended in 200 μL of red blood lysis buffer (155 mM NH_4_Cl, 10 mM NaHCO_3_), pH 7.2. Cells were washed again with 200 μL 1× PBS and resuspended in 50 μL of a 1:50 dilution of Fc block (CD16/CD32, 2.4G2) in fluorescence-activated cell sorting (FACS) buffer (0.1% sodium azide in 1× PBS). After incubation on ice for 15 to 20 min, cells were washed with 150 μL of FACS buffer and resuspended in 50 μL of the appropriate antibody mixture for each strain. Following 45 to 60 min of coincubation on ice, samples were washed with FACS buffer and resuspended in 200 μL of FACS buffer for flow cytometry. Cell surface antibodies anti-CD45 antibody (30-F11 BUV395), anti-Thy1.2 antibody (53-2.1 PE-Cy7), anti-CD4 antibody (RM4-4 FITC), and anti-CD8α antibody (53-6.7 Pacific blue) were used to confirm CD4^+^ or CD8^+^ T cell depletion.

### Lung processing.

Single-cell suspensions of pulmonary cells were prepared for flow cytometric analysis as previously described (36). In brief, lung tissue was minced in 5 mL of 1× PBS containing 3 mg/mL collagenase type IV (Worthington). Samples were incubated at 37°C for 45 min and washed with 1× PBS three times. After digestion, residual red blood cells (RBCs) were removed using RBC lysis buffer (155 mM NH_4_Cl and 10 mM NaHCO_3_, PH 7.2). Lung cell suspensions were used for flow cytometry. Lung single-cell suspensions were stained for monocytes (CD45 [30-F11BUV395], CD11b [M1/70 peridinin chlorophyll protein {PerCP} Cy5.5], and Ly6C [AL-21 PE]), Mo-DCs (CD45 [30-F11 BUV395], CD11b [M1/70 PerCP Cy5.5], Ly6C [AL-21 PE], CD11c [HL3 BV510], and major histocompatibility complex [MHC] class II I-A/I-E [M5/11.415.2 BV711]), neutrophils (CD45 [30-F11 allophycocyanin {APC}-Cy7], CD11b [M1/70 PerCP Cy5.5], Ly6C [AL-21 PE], and Ly6G [1A8 APC]), CD4 T cells (CD45 [30-F11 BUV395] and CD4 [RM4-5 BV421]), and CD8 T cells (CD45 [30-F11 BUV395] and CD8α [53-6.7 BV711]). All antibodies used for lung staining were from BD Biosciences. All samples were analyzed using the BD LSR Fortessa flow cytometer and FlowJo software (Tree Star, Inc.).

### Intracellular cytokine staining of T cells harvested in BALF and flow cytometry.

For analyzing host immune responses, bronchoalveolar lavage fluid (BALF) samples were harvested at the endpoint after inoculation. BALF was collected in 3 mL of 1× PBS buffer using a catheter inserted into the trachea of the animal posteuthanasia, and airway-infiltrating cells were lavaged with ~1 mL of 1× PBS at a time to a total volume of 5 mL. RBCs were removed using RBC lysis buffer. BALF cells were then plated in a 96-well round-bottom plate and restimulated using BD leukocyte activation cocktail containing BD GolgiPlug (BD Biosciences) according to the manufacturer’s instructions. Six hours after activation, BALF cells were surface stained with fluorescently labeled antibodies against Thy1.2, CD4, and CD8. Samples were fixed in 1% paraformaldehyde overnight. Prior to intracellular staining, the samples were permeabilized with 1× BD Perm/Wash buffer according to the manufacturer’s instructions. Intracellular cytokine staining (ICCS) was done using fluorescently labeled antibodies against IFN-γ, IL-17A, tumor necrosis factor alpha (TNF-α), and IL-13 diluted in 1× BD Perm/Wash buffer for 30 min on ice. Samples were immediately washed and analyzed by flow cytometry as described below. BALF samples were cell surface stained for T cells with Thy1.2 (53-2.1 PE-Cy7), CD4 (RM4-5 BV421), and CD8α (53-6.7 BV711), and ICCS was used for IFN-γ (XMG1.2 PE), IL-17A (eBio17B7 APC), TNF-α (MP6-XT22 BV711), and IL-13 (eBio13A FITC), following standard procedures. Most antibodies and reagents for cell surface staining and ICCS were from BD Biosciences, except for IL-17A and IL-13, which were obtained from eBioscience, Inc.

### CD4^+^ T cell isolation and CD4^+^ T cell recall response.

Antigen-presenting cells were prepared from the spleens of syngeneic, uninfected donor mice. Splenic cell suspensions were depleted of T cells by antibody complement-mediated lysis. Splenic cells were incubated with anti-Thy1.2 antibodies and rabbit complement (Low Tox; Cedarlane Labs) at 37°C for 1 h. Lung-draining lymph nodes (mLN) were collected and placed in 10 mL of 1× PBS. Total lymphocyte cell suspensions were prepared by gently releasing the cells into the 1× PBS by applying pressure to the lymph nodes with the frosted ends of two glass slides. Repeated pressure was applied until the tissue was reduced to the smallest size possible. Samples were collected and processed in the same way individually. For CD4 T cell isolation, individual samples from each group were pooled (5 mice). CD4^+^ T cells were purified using a negative-sorting CD4^+^ isolation kit (Miltenyi Biotec, Inc.). CD4^+^ T cell isolation was done following the manufacturer’s instructions, and the isolated cells were consistently found to be >90% pure, as assessed by flow cytometry. Purified CD4^+^ T cells (2 × 10^5^) were cultured with T cell-depleted antigen-presenting cells (3 × 10^5^) in RPMI medium containing 10% fetal calf serum (FCS), penicillin-streptomycin (2,200 U/mL, Gibco), and gentamicin sulfate solution (1 mg/mL). The cultures were plated in flat-bottom 96-well plates and incubated at 37°C with 5% CO_2_ for 72 h. To measure Cryptococcus-specific CD4^+^ T cell responses, CD4-antigen-presenting cell cultures were incubated with sonicated (Qsonica sonicator Q55) H99 yeasts as a source of fungal antigens. The amount of antigen used was adjusted to a multiplicity of infection of 1:1.5 (antigen-presenting cell/yeast ratio). The fungal growth inhibitor voriconazole was used at a final concentration of 0.5 mg/mL to prevent any fungal cell outgrowth during the culture period. After 72 h of culture at 37°C with 5% CO_2_, supernatants were collected for cytokine analysis by enzyme-linked immunosorbent assay (ELISA) (IL-2, BD OptEIA; IL-17A and IFN-γ, ThermoFisher) following the manufacturer’s instructions.
