# Supplementary material for: Development of a Heat-Killed fbp1 Mutant Strain as a Therapeutic Agent To Treat Invasive Cryptococcus Infection
Source: Microbiol Spectr. 2023 Jan 31;11(2):e04955-22. doi: 10.1128/spectrum.04955-22 (PMC10101017; doi:10.1128/spectrum.04955-22)
Supplement: Supplemental file 1 — Fig. S1 and S2. Download spectrum.04955-22-s0001.pdf, PDF file, 0.7 MB [file spectrum.04955-22-s0001.pdf]

Supplementary materials

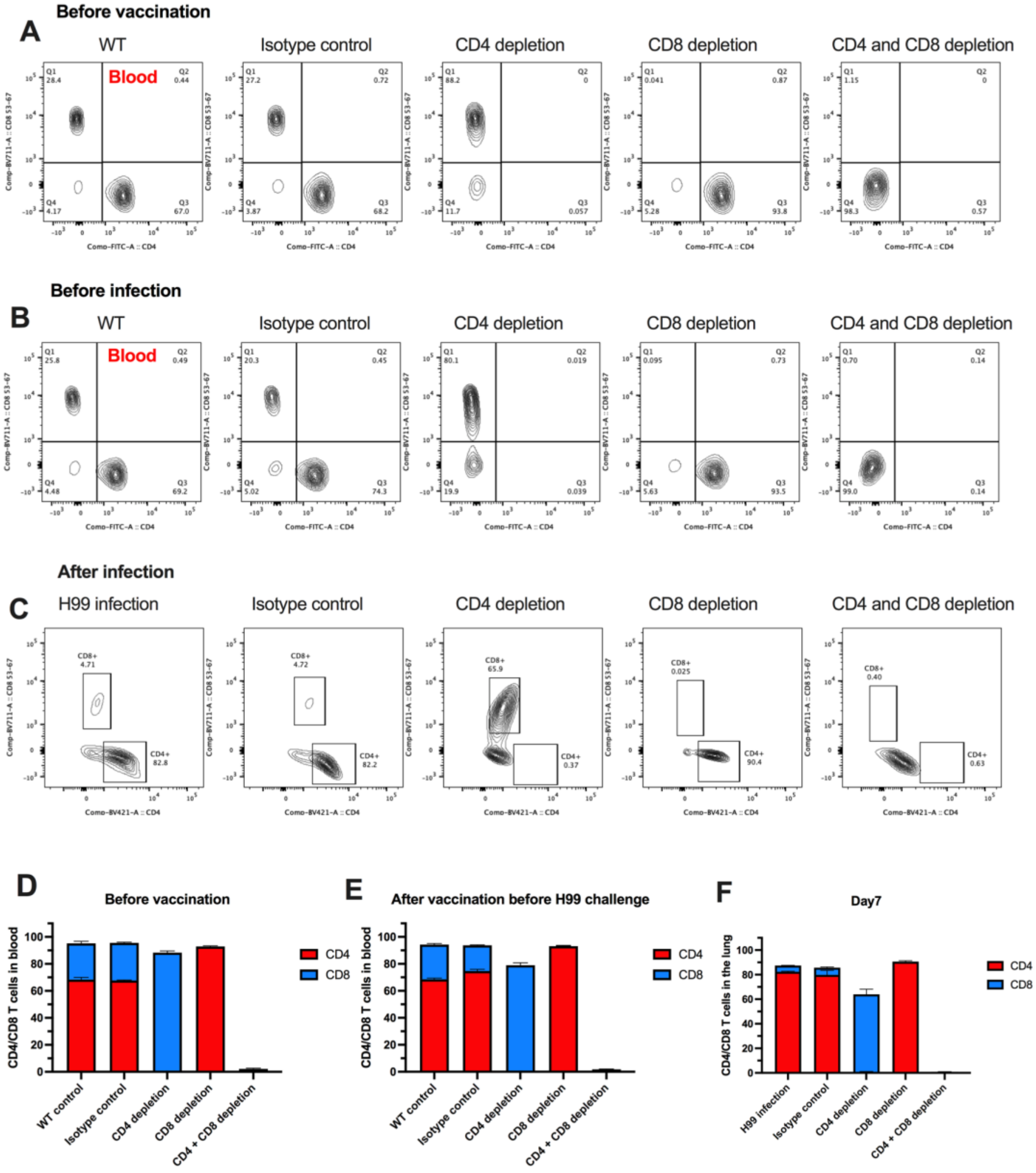

Figure S1. (A and B) Detection of $CD4^+$ and $CD8^+$ T cells by flow cytometry prior to first vaccination (day -43**) (A)** and prior to challenge infection (day -1) **(B)** in mice injected with either CD4 antibody, CD8 antibody or isotype antibody. Representative FACS plots of $CD4^+$ and $CD8^+$ T cells in blood samples of $CD4^+$ T cells depleted mice, $CD8^+$ T cells depleted mice, double depleted mice, isotype control treated mice and wild type infection only mice were shown. **(C)**

Efficient depletion of $CD4^{+}$ and $CD8^{+}$ T cells in mice was confirmed by flow cytometry at day 7 post-challenge. Representative FACS plots of $CD4^{+}$ and $CD8^{+}$ T cells from lung tissues of $CD4^{+}$ T cells depleted mice, $CD8^{+}$ T cells depleted mice, double depleted mice, isotype control mice and wildtype infection only mice were shown. Each cell population was identified as $CD45^{+}$, 4',6-diamidino-2-phenylindole (DAPI)-negative live leukocytes. $CD4^{+}$ T cells was gate as $CD11b^{-}$, $CD4^{+}$, $CD8^{-}$; and $CD8^{+}$ T cells were gated as $CD11b^{-}$, $CD4^{-}$, $CD8^{+}$. **(D-F)** Percentages of $CD4^{+}$ and $CD8^{+}$ T cell in mouse blood at different times in the course of vaccination study: before vaccination **(D)**, before H99 challenge **(E)**, and at day 7 post-challenge **(F)**.

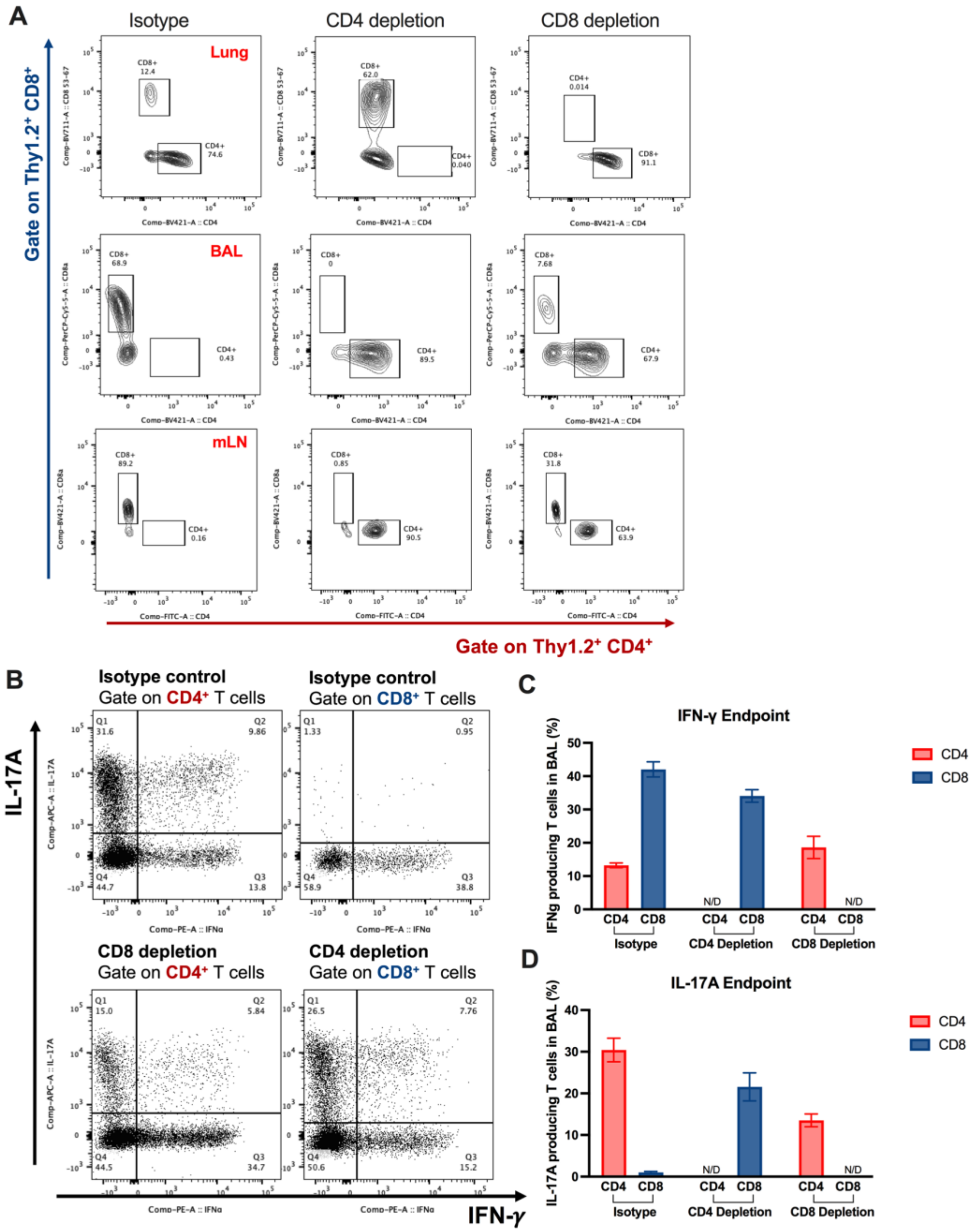

Figure S2. (A) At the endpoint of experiment (day 70), representative FACS plots of $CD4^+$ T cells and $CD8^+$ T cells in the lung, BALF, lung-draining lymph nodes in isotype control mice (left), $CD4^+$ T cells depleted mice (middle), and $CD8^+$ T cells depleted mice (right). **(B)** Representative FACS plots of cytokine production gate on $CD4^+$ T cells or $CD8^+$ T cells in isotype control mice

(top), CD4 depleted mice (bottom), and CD8 depleted mice (bottom) at endpoint. **(C and D)** Cytokine expression at endpoint analyzed by ICCS. Plots of cytokine production in $CD4^+$ T cells gated as $Thy1.2^+$ $CD4^+$ $CD8^-$ T cells. Plots of cytokine production in $CD8^+$ T cells gated as $Thy1.2^+$ $CD4^-$ $CD8^+$ T cells. The frequencies of IFN-γ **(C)**, IL-17A **(D)** producing $CD4^+$ or $CD8^+$ in BALF were analyzed as shown in panel.
